# Development of a Method for the Determination of Chromium and Cadmium in Tannery Wastewater Using Laser-Induced Breakdown Spectroscopy

**DOI:** 10.1155/2012/823016

**Published:** 2012-02-29

**Authors:** Mahwish Bukhari, M. Ali Awan, Ishtiaq A. Qazi, M. Anwar Baig

**Affiliations:** Institute of Environmental Science and Engineering, School of Civil and Environmental Engineering, National University of Sciences and Technology, Sector H-12, Islamabad 44000, Pakistan

## Abstract

This paper illustrates systematic development of a convenient analytical method for the determination of chromium and cadmium in tannery wastewater using laser-induced breakdown spectroscopy (LIBS). A new approach was developed by which liquid was converted into solid phase sample surface using absorption paper for subsequent LIBS analysis. The optimized values of LIBS parameters were 146.7 mJ for chromium and 89.5 mJ for cadmium (laser pulse energy), 4.5 *μ*s (delay time), 70 mm (lens to sample surface distance), and 7 mm (light collection system to sample surface distance). Optimized values of LIBS parameters demonstrated strong spectrum lines for each metal keeping the background noise at minimum level. The new method of preparing metal standards on absorption papers exhibited calibration curves with good linearity with correlation coefficients, *R^2^* in the range of 0.992 to 0.998. The developed method was tested on real tannery wastewater samples for determination of chromium and cadmium.

## 1. Introduction

Leather industry is one of the major industrial sectors in Pakistan that contributes substantially towards national economy [1]. There are 725 tanneries in Pakistan and most of them employ chrome tanning because of the rapid processing, low cost, and better quality of the finished leather products [2].

The leather tanneries generate different types of wastes out of which toxic wastewater and solid waste pose significant environmental challenges [3]. Tannery wastewater generally contains high concentrations of organic matter, solids, sulfates, sulfides, and chromium and exhibit very high levels of chemical oxygen demand and biochemical oxygen demand [4].

Most of the tannery units discharge untreated effluents directly into recipient water bodies or into open land. High amounts of chromium were observed in the effluents discharged from chrome tanning process [5]. The contamination levels of chromium and cadmium in tannery wastewater are observed to be several hundred times higher as compared to the values recommended in the National Environmental Quality Standards (NEQSs) set by the Government of Pakistan [6]. The uncontrolled discharge of untreated tannery wastewater causes health problems to tannery workers and the local communities. Heavy metals such as chromium and cadmium are toxic even at low concentrations; a gradual buildup of these toxins in the human bodies may cause long-term health damages [7]. Hence, the regular monitoring of these metals in the tannery effluents is of utmost importance in order to suggest remedial measures.

The heavy metal analysis in the industrial effluents is generally carried out by conventional analytical techniques like atomic absorption spectroscopy (AAS) and UV-Visible spectrophotometry. Both these analytical techniques require time-consuming sample preparation protocols [5, 8]. The present study aims to establish an alternate instrumental methodology that uses the direct introduction and analysis of tannery wastewater (after rapid solidification) without undergoing time-consuming sample preparation steps. A laser-induced breakdown spectroscopy (LIBS) could be one such technique as it has been applied successfully in a number of studies for the determination of elemental concentrations of a wide range of samples [9].

LIBS technique is a type of atomic emission spectroscopy that focuses on intense pulsed laser beam onto the sample, resulting in the dissociation of the sample in a fast growing plasma cloud. The dissociated sample atoms and ions (plasma) generate spectra comprising of atomic emission lines corresponding to the atoms present in the plasma (sample) [10]. Hence, LIBS analysis gives information about the elements in the form of emission lines located at specific wavelengths. LIBS has rapidly developed into an important analytical technique with the capability of detecting several elements in the sample simultaneously [10]. The spectra may further be explored to determine the concentration of elements present in the samples. The most practical approach is to measure the spectral line intensities in relation to known calibration standards [11]. LIBS is generally used for the rapid elemental analysis of solid samples. The recent applications include determination of sulfur and chloride in reinforced concrete [12, 13] and lead in solidified paint sample [14]. The present study describes the systematic development and optimization of a method for the quantification of two environmentally significant heavy metals; chromium and cadmium in tannery wastewater.

## 2. Materials and Methods

The metal salts (chromium chloride and cadmium nitrate 99%) were purchased from Merck (Germany). ALBET (Germany) absorption papers for Petri (size 47 mm) were used for solidification of liquid standards and tannery wastewater samples.

**Figure 1:**
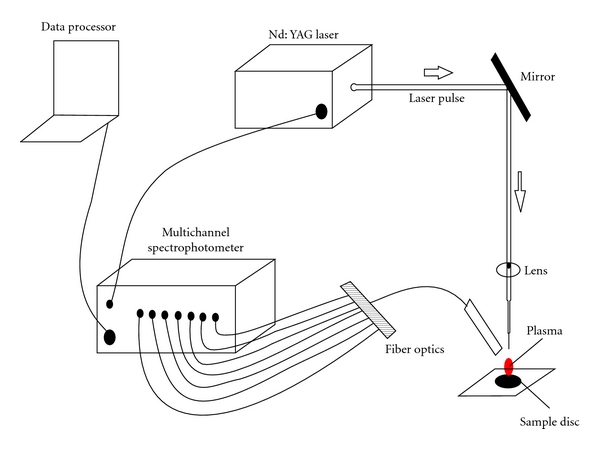
Schematic diagram of an LIBS2500plus system.

The analytical instruments used in this study include Laser-induced Breakdown Spectrometer (LIBS); Ocean Optics LIBS2500plus, USA, and Flame Atomic Absorption Spectrometer; Varian AA 240, USA. The LIBS2500plus is a high-resolution instrument that allows spectral analysis in the range 200–980 nm. Q-switched Nd:YAG laser operates at 1064 nm wavelength and delivers maximum pulse energy of 200 mJ with a pulse width of 10 ns and runs at a 10 Hz pulse repetition rate. A high-intensity, 10 nanosecond-wide laser pulse beam is focused on the sample surface. The instrument has seven spectrometer modules to provide high resolution with a gated charge-coupled detector (CCD) having 14,336 pixels for simultaneous recording of the spectra. OOILIBS software with spectral-saving and data-logging capabilities displays and identifies the emission spectrum. The schematic of a LIBS2500plus system is shown in Figure 1.

The LIBS2500plus has the capability of analyzing of solid samples only. Therefore, liquid-phase samples and standards were converted to into solid phase for subsequent analysis on LIBS.

### 2.1. Solidification of the Liquid Metal Standards

Solidification of liquid chromium and cadmium standards was achieved by introducing small amount of liquid standard on absorption paper followed by ambient drying. The absorption papers are made of pure cellulose and have an open pore structure for a high degree of liquid absorption in homogenous fashion (uniform spread). In the present study, measured quantities of liquid standards and real samples were poured on these papers to obtain solidified surface for LIBS analysis.

Chromium and cadmium standards were prepared by dissolving calculated amount (mass) of respective metal salts in 1 mL of pure methanol in clean test tube. 0.5 mL of the solution was poured on an absorption paper. The paper was left at ambient temperature for drying for subsequent analysis on LIBS. Methanol solvent was selected due to its high volatility and rapid dying. For the formation of calibration curves, chromium and cadmium standards were prepared using the above mentioned procedure.

### 2.2. Optimization of LIBS Parameters

The LIBS system was optimized for four parameters; CCD delay time, laser pulse energy, lens to surface distance, and light collection system (fiber optic) to surface distance in order to obtain strong spectrum lines for each metal keeping the background noise at a minimum level. The optimization was carried out using the solidified metal standards. Chromium and cadmium calibration standards were analyzed (in replicate) by LIBS under optimized instrument parameters.

### 2.3. Tannery Wastewater Sampling

Five wastewater samples were collected from tanneries located along the Lahore-Kasoor road and Samberial road, Sialkot. Two samples were collected directly from tanning drums and the remaining three from the main drains in 500 mL polyethylene bottles and acidified with HNO_3_ till pH dropped below 2. 0.5 mL of the sample was poured on the absorption paper for solidification. The dried samples were analyzed for the metal contents by LIBS under optimized parameters.

The metal (chromium and cadmium) contents of selected samples were also determined by the conventional standard analytical technique, flame atomic absorption spectrometry (FAAS). FAAS calibration curves were prepared using five chromium and cadmium standards of different concentrations.

## 3. Results and Discussion

### 3.1. Solidification Metals Standards and Analysis on LIBS

**Figure 2:**
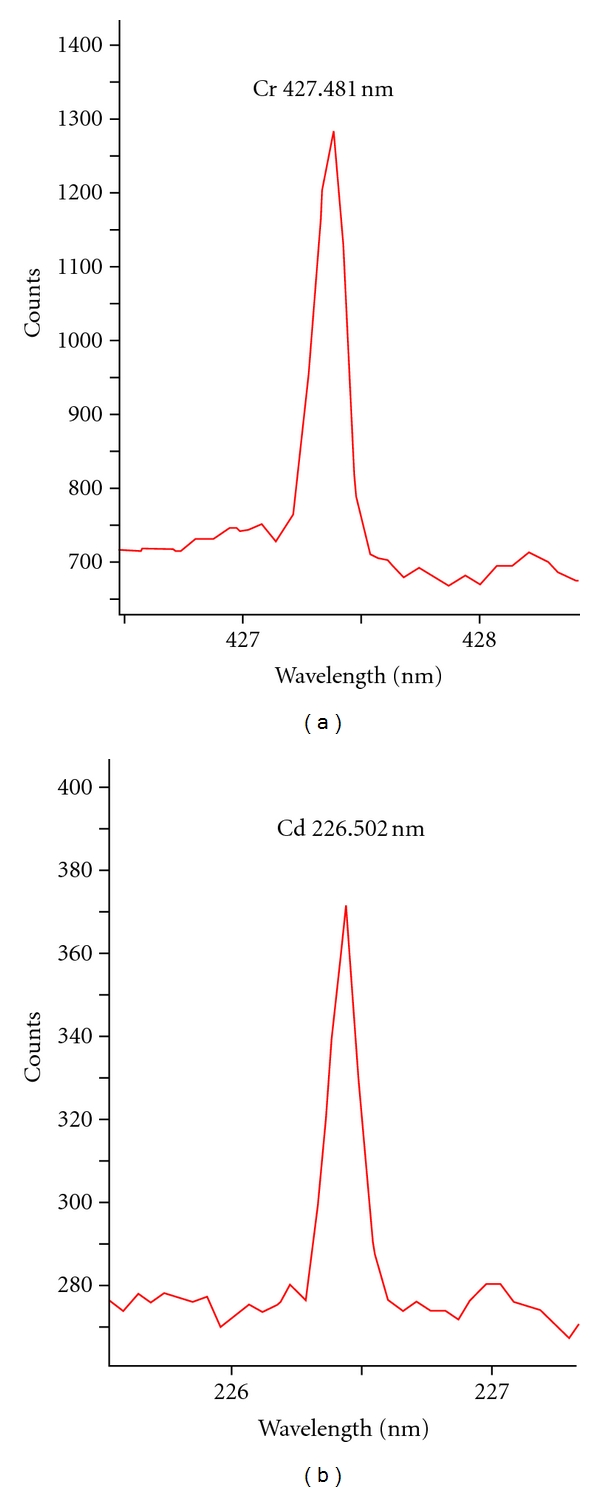
LIBS spectra of chromium and cadmium standards showing emission lines at 427.481 nm and 226.502 nm, respectively.

**Figure 3:**
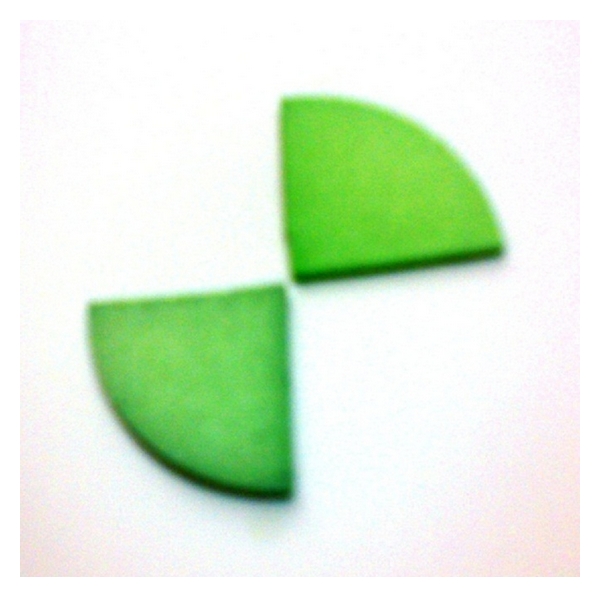
Pieces of absorption paper showing homogenous spread of chromium standard.

The solidified metal standards were used to select spectral lines of maximum emissivity for chromium and cadmium.  Figure 2 shows LIBS spectra with a prominent emission lines at wavelengths 427.481 nm and 226.502 nm for chromium and cadmium, respectively. The emission line for each metal was confirmed using National Institute of Standards and Technology (NIST) electronic database. The spectral data was recorded in terms of  “signal-to-background ratio (SBR)” in order to reduce effect of background noise due to matrix effect. The analysis of solidified metal standard at different places exhibited precise SBR values (RSD < 2%) that proved the uniform distribution of metal on absorption paper, see Figure 3. The developed solidification method is much simpler (single step/direct) as compare to the reported technique involving calcium oxide for liquid-to-solid matrix conversion [15].

### 3.2. Optimization of LIBS Parameters

LIBS like other analytical techniques requires optimization of instrumental parameters for the generation of authentic quantitative results. The important parameters which can influence the sensitivity of LIBS system include laser pulse energy, lens-to-surface distance (LTSD), CCD delay time [16], and light collection system (fiber optic) to surface distance. The light emitted from plasma is spectrally resolved and analyzed for the identification and concentration of the trace elements present in the sample [17].

**Figure 4:**
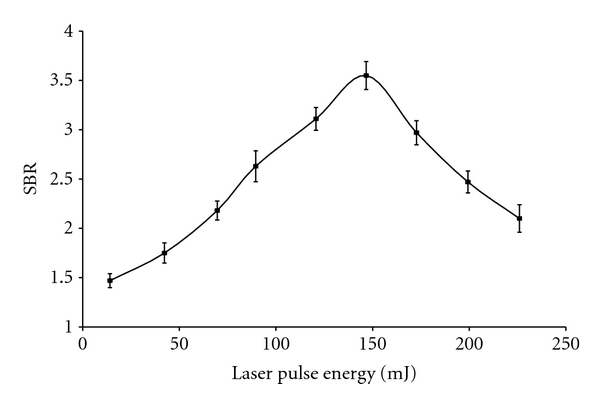
Signal intensity (SBR) of chromium (427.481 nm) versus laser energy (mJ). Error bars denote the 95% confidence level and are based on replicate (10) measurements.

The laser source of an LIBS system vaporizes, atomizes, and excites the sample material. In the present study, signal intensities were examined as a function of laser pulse energies in the range 10 to 230 mJ in order to determine optimum pulse energy at lens-to-surface distance 75 mm and CCD delay time 4.5 *μ*s.  Figure 4 shows signal intensity in terms of SBR versus laser pulse energy for chromium standard at 427.481 nm. LIBS signals increased proportionally to the laser pulse energy till the plasma density became too high where the phenomenon of self-absorption started by the metals [11]. The dense plasma formed by leading laser pulse starts absorbing energy from the later part of the laser pulse which leads to higher continuum emission and lowers the analyte signal [18]. A reasonable line intensity and precision was obtained at 146.7 mJ for chromium and 89.5 mJ for cadmium. Hence, these values of laser pulse energies were selected for subsequent analyses.

Analytical of measurements by an LIBS system may greatly vary by changing distance between the focusing lens and the sample surface [19]. A change of the LTSD of a few millimeters can affect the intensity of the analyte emission line/s. Therefore, it is important to find out optimized value LTDS where analyte signal intestines are maximum. The highest maximum signal intensity (SBR) was obtained at 75 mm of LTSD and was thus selected for consequent analyses for better accuracy and precision of the results.

**Figure 5:**
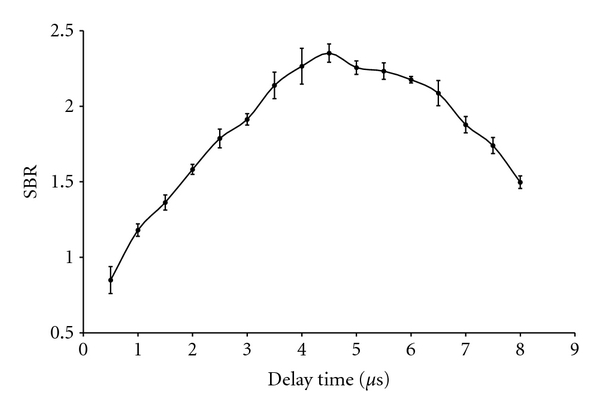
Signal intensity (SBR) versus delay time for chromium standard at 427.481 nm. Error bars denote the 95% confidence level and are based on replicate (10) measurements.

A series of measurements on chromium and cadmium standards were carried out to determine the optimum delay time for maximum LIBS signal intensities (SBR). Delay time is the time between the firing of the laser pulse and the opening of the camera shutter to collect the emissions from the sample surface [10].  Figure 5 shows signal intensity versus delay time trend for chromium standard at 427.481 nm. The intensity of the emission line increased with time and attained maximum value at a delay time of 4.5 *μ*s which agreed with the reported value [20]. Beyond 4.5 *μ*s, the intensity of emission line decreased because the plasma probably might have started cooling down.

Distance of light collection system (fiber optic) from sample surface was also optimized to ensure the appropriate collection of emission lines from the sample plasma because a change in the distance between fiber optic and plasma (sample surface) might affect LIBS signal intensity. The optical fiber is pointed at angle 45° relative to sample surface. The optimized value for the distance from sample surface to fiber optic was found to be 7 mm for both metals.

### 3.3. Calibration Curves

**Figure 6:**
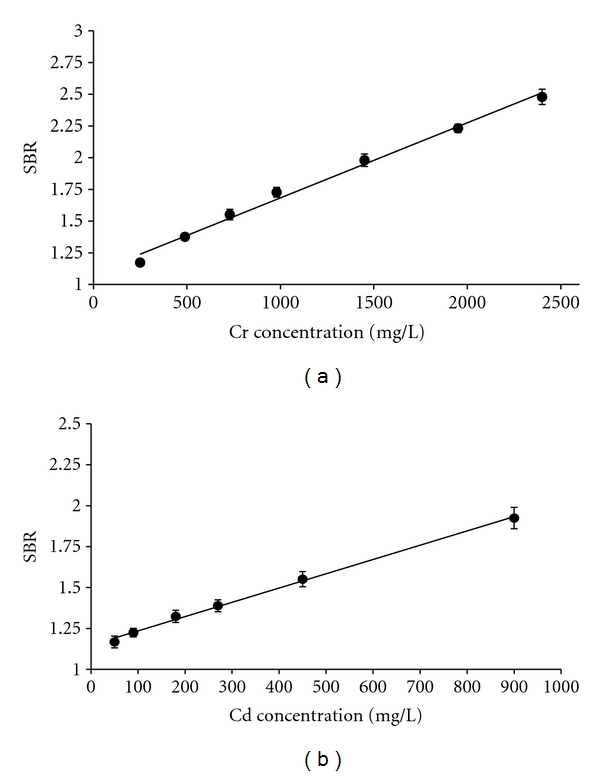
Calibration curve; concentration versus signal intensity (SBR) for Cr (427.481 nm) and Cd (226.502 nm). Error bars denote the 95% CI and are based on replicate (10) measurements.

Conducting a calibration study before quantitative analysis is a prerequisite for any analytical technique including LIBS.  Figure 6 exhibits calibration curves constructed by running chromium and cadmium standards at the wavelengths of 427.481 nm and 226.502 nm, respectively. The precision among the values of each calibration data point (replicate SBR) was calculated with the help of 95% confidence intervals. Error bars shown in Figure 6 denote precision in the form of 95% CI and are based on replicate (10) measurements. A linear regression approach was used to fit the appropriate trend lines to LIBS data obtained. All calibration curves demonstrated good linearity with correlation coefficients, *R* ^2^ of 0.992 for chromium and 0.998 for cadmium, respectively.

The calculation of the limit of detection (LOD) is an important part of any calibration study. LOD is the lowest concentration that can be detected by an analytical method. In this study, the LODs of two metals were determined by using the calibration data; *y*-intercept and standard deviation of the regression [21], see equation below:

(1) $$y_{\text{LOD}}=y_{\text{intercept}}+3{\left[\frac{\sum \left(y_{i}-\overset{®}{y_{i}}\right)}{n-2}\right]}^{1/2},$$

where $\left(y_{i}-\overset{®}{y_{i}}\right)$ is linear regression *y*-residual and *n* is number of calibration data points. The calibration curve equations were then used to convert the signal *y*_LOD_ to LODs in concentration units. The values of LOD for chromium and cadmium were 230 mg/L and 46 mg/L. Since the calibration curves were constructed in the high-concentration range therefore, the derived LODs values appear high, but still these are appropriate for the analysis of thick tannery effluents being discharged.

### 3.4. Tannery Wastewater Analysis

**Figure 7:**
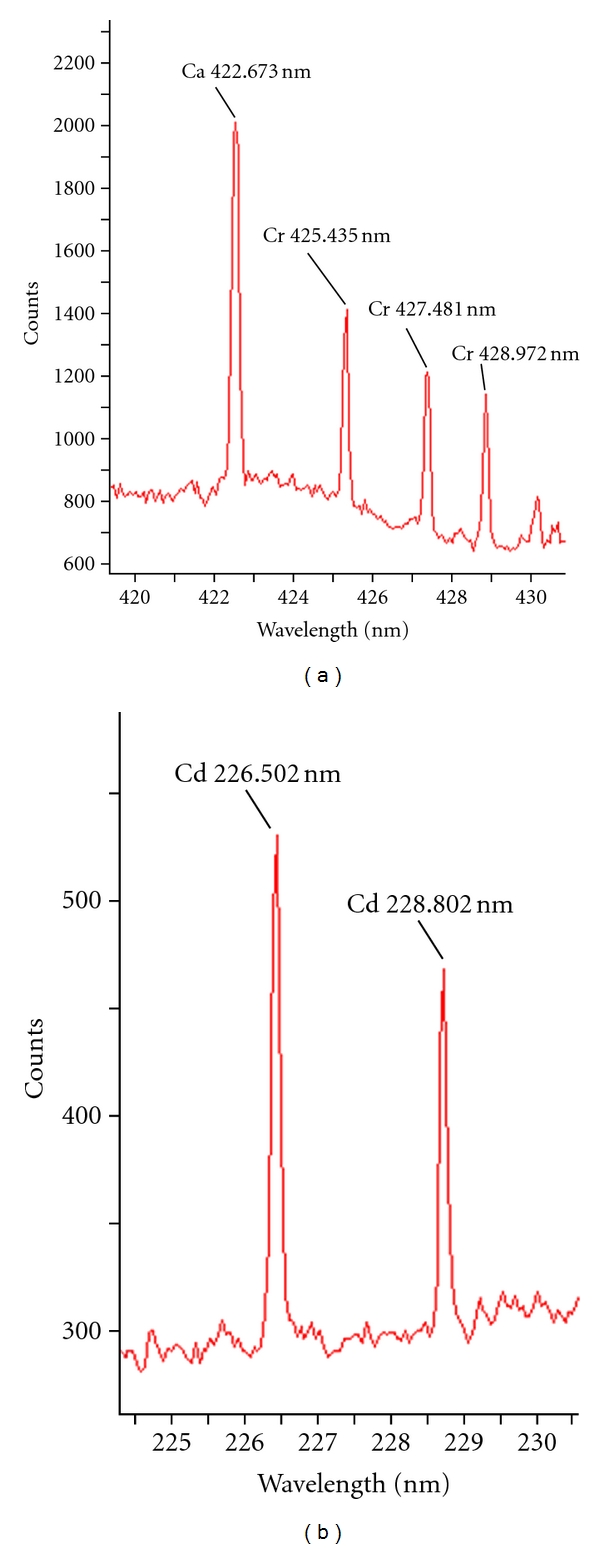
LIBS spectra showing chromium and cadmium lines in Sialkot tannery wastewater sample.

The calibration equations obtained by the LIBS-developed method were used to estimate the levels of chromium and cadmium in tannery wastewater samples.  Figure 7 exhibits LIBS spectra showing chromium and cadmium lines in Sialkot tannery wastewater sample. For quantification purposes, average spectra of 20 laser shots were recorded for each metal present in sample under optimized LIBS parameters. The metals were determined in units of mg/L in order to have a comparison with the analysis by atomic absorption spectrophotometry. LIBS analysis showed (Table 1) that the concentration of chromium in Sample-1 and 2 was above 2000 mg/L because these two samples were collected directly from the tanning drums during tanning process; Whereas the concentration of chromium in other three samples was comparatively low due to dilution of the tanning wastewater in the main and side drains. The levels of chromium observed using developed LIBS method were found to be comparable with those reported previously [5].

On the other hand, the level of cadmium in all samples (except sample-4) was around 500 mg/L. The probable reason of somewhat uniform levels of cadmium in all samples might be the addition of effluents from leather dying sections into the same main and side drains. Such effluents carried cadmium-containing dyes and pigments hence its level remained high in all the samples.

Table 1 gives a comparison of the quantitative analyses made both by the developed LIBS method and FAAS. It is evident that there is a good correlation between the results of these two different analytical techniques. Both the techniques determined concentrations of chromium and cadmium in the same degree of magnitude and agreed reasonably well with each other. This is a worthy indication that our developed LIBS method would effectively be used for the rapid analysis of chromium and cadmium in an industrial wastewater.

## 4. Conclusions

A convenient LIBS method has been developed for the detection of heavy metals (chromium and cadmium) in tannery wastewater samples based on transformation of liquid-phase samples to solid phase on absorption papers. The use of absorption papers ensured homogeneous spread of metal standards and samples. The new method of preparing metal standards on absorption papers exhibited calibration curves with good linearity over the concentration range under investigation. The concentration of two metals determined by the developed LIBS method was verified against a conventional analytical technique, flame atomic absorption spectroscopy (FAAS). The results were comparable and proved that LIBS has a considerable potential for the qualitative and quantitative analysis of metals present in tannery wastewater samples.

## Figures and Tables

**Table 1:** Comparison of chromium and cadmium concentrations determined both by LIBS and FAAS in selected Tannery wastewater samples.

Sample	Cr ppm ± SD		Cd ppm ± SD	
	LIBS	FAAS	LIBS	FAAS
S-1	2192 ± 37	2245 ± 1.1	519 ± 15	533 ± 0.5
S-2	2225 ± 29	2269 ± 1.4	563 ± 17	575 ± 0.3
S-3	495 ± 13	459 ± 0.4	590 ± 12	530 ± 0.2
S-4	249 ± 7	278 ± 0.2	867 ± 13	832 ± 0.4
S-5	331 ± 9	304 ± 0.4	591 ± 7	570 ± 0.3
